# Supplementary figures and images for: Spatial and temporal variation in the occurrence of bottlenose dolphins in the Chesapeake Bay, USA, using citizen science sighting data
Source: PLoS One. 2021 May 18;16(5):e0251637. doi: 10.1371/journal.pone.0251637 (PMC8130941; doi:10.1371/journal.pone.0251637)

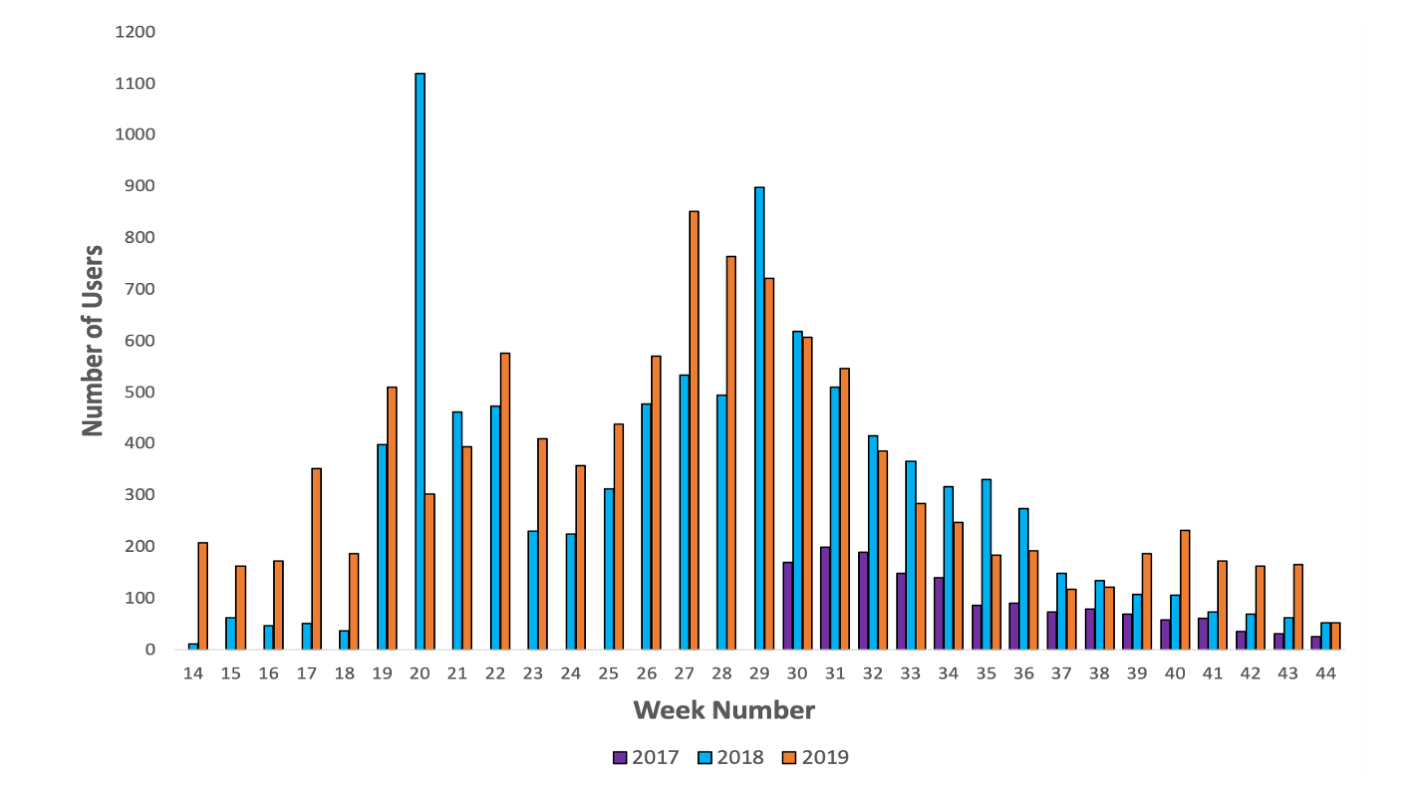

Supplement: S1 Fig — (TIF) [file pone.0251637.s001.tif]
