# Supplementary material for: Spatial and temporal variation in the occurrence of bottlenose dolphins in the Chesapeake Bay, USA, using citizen science sighting data
Source: PLoS One. 2021 May 18;16(5):e0251637. doi: 10.1371/journal.pone.0251637 (PMC8130941; doi:10.1371/journal.pone.0251637)
Supplement: S1 File — (DOCX) [file pone.0251637.s002.docx]

**
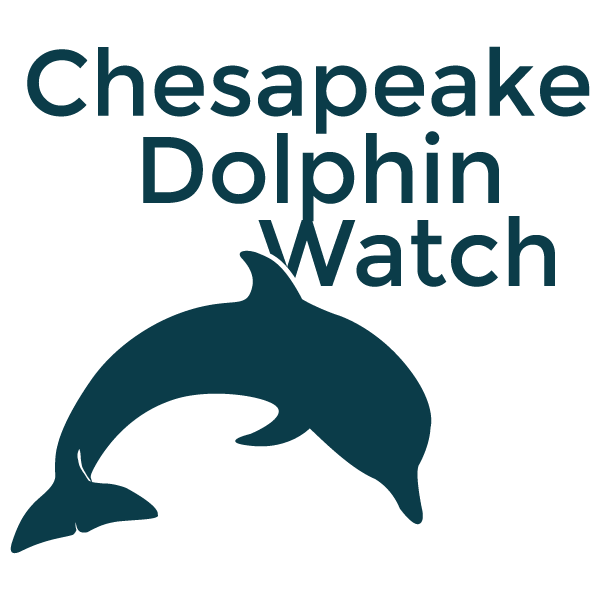
**

**DolphinWatch Sighting Report Confirmation Criteria**

All sightings entered into the app are automatically set to ‘unconfirmed’ status by the software. When a sighting has been reviewed by a staff member it is confirmed under the following criteria:

-there is a photo or video of the encounter to accompany the sighting details provided *and* the sightings plots in the water

or

-there is a description of the sighting *and* the sighting was located in a portion of the Bay or its tributaries where previous sightings have been documented in the same time of year

or

- the user is part of a small list of “trusted application users” (n=14) who have previously demonstrated competence in identifying bottlenose dolphin in Chesapeake Bay multiple times for Chesapeake DolphinWatch through detailed descriptions *and* images (photos/videos)

___________________________________________________________________________

Note: If a sighting plots on land and contains a photo/video and/or written description, staff will make an attempt to correct the information by emailing the user to get corrected data. If the sighting plots on land and contains no other details staff email the user to get an updated location. If the location cannot be corrected it remains unconfirmed for the season and is ‘rejected’ at the year’s end. If the description of the location, time, group size, etc does not match the description written by the user, staff may email the user to clarify the data. If the sighting occurs in an area not previously known to have dolphin sightings and does not have a photo/video to corroborate, it remains unconfirmed, unless the user sends enough additional information (sometimes just a photo/video) to confirm the sighting with confidence.

Excerpt from the Chesapeake DolphinWatch Handbook (last updated August, 2020):

Once staff have reviewed the sighting, and possibly contacted the user who reported the sighting, it is marked confirmed or rejected by staff. If a user did not enter a description or attach an image to their sighting report they receive an automatic email request to do so from the app. Sightings which do not have a description or image are left unconfirmed. Sightings which are in the water and have a description or image are generally confirmed, even if they are outside of Chesapeake Bay. When a sighting report appears on land and do not have a description of the sighting location staff will email the user to ask to correct or clarify the sighting location. When a sighting report appears on land but the description contains location information staff edit the location to match the description provided and note they did so in the notes section of the reporting box. Sightings which are in the water but occur in an area not known to have dolphins and do not have an image of the animals are left unconfirmed and staff email for more information. Locations such as rivers north of the Chester River, like Patapsco River, or locations in the main stem above Hart-Miller Island, in/around Baltimore, north of Baltimore do not typically report dolphins so these are reviewed meticulously.
