# Supplementary material for: Spatial and temporal variation in the occurrence of bottlenose dolphins in the Chesapeake Bay, USA, using citizen science sighting data
Source: PLoS One. 2021 May 18;16(5):e0251637. doi: 10.1371/journal.pone.0251637 (PMC8130941; doi:10.1371/journal.pone.0251637)
Supplement: Attachment — Submitted filename: PONE-D-20-27115_Review.docx [file pone.0251637.s003.docx]

**PONE-D-20-27115 Review comments: Oct 2020**

**General Comment**

Authors present data on dolphin sightings in Chesapeake Bay, USA using citizen science (CS) data collected over three years. The research provides an important baseline dataset to inform conservation management and impact assessment into the future. It is recommended that the authors provide additional information on the observer bias and effort from CS sightings and how this affected the results. The paper is well written. Some sections could use some restructuring and clarity. The analysis supports the objectives and results. Some justification could be further provided on selection of variables i.e. why was depth not considered? The author is encouraged to define the seasons and survey period more clearly throughout the manuscript. Please refer to sections below for specific comments. It is recommended that this paper is accepted with minor revisions.

**Title:** Can you insert Chesapeake Bay, USA?

**Abstract**

- To avoid repetition you could remove ‘to determine relative occurrence throughout the estuary’
- Were CS sightings recorded all year round? Please clarify
- Was the acoustics data collected opportunistically, through CS as well?

**Introduction**

- Line 71: Insert United States (U.S.), same for first use of USA.
- Line 97: Results/ sample size/No. sightings should be reported in the results section, not introduction

**Methods**

- Line 145: remove ‘their’
- Line 153: was there effort bias in different months i.e. sunny days in summer vs cold winter days. How did number of observers vary? Was effort recorded when no sightings were recorded?
- Line 155: assume this is location of the observer, not the dolphin. Did you calculate an error range, what was the distance of dolphins from observer? Was there other species in the Bay? Were photographs used to confirm sighting records/species. Was there any training completed for CS to have a level of competency when recording sightings data, species, group size etc?
- Line 177: Did you consider duplicates of the same dolphin group in a day, i.e. sightings of the same group in different areas >30m apart
- Line 192: perhaps add, during the 6-month period that the loggers were deployed each year. How did you consider observer bias outside of the 6-month period the loggers were deployed?

**Results**

- Line 277: what was the criteria for ‘confirming’ or excluding the sightings data?
- Line 279: Please define whether you are talking about inter or intra seasonal variation.
- I suggest restructuring text and separating the paragraphs here to report results for a) inter-seasonal variation b) intra-seasonal variation within years including peak numbers and c) spatial variation inter and intra seasonal.
- Line 310: were the CS land based of boat based? Assuming that the location of the observer was recorded, not the dolphins, what is your confidence for the location of sightings?
- Line 314: was highest sightings in this area because of concentrated effort?
- Can you assess statistic difference between no of sightings in different areas and if the occurrence was statistically different in different zones, insert p-value?

**Discussion**

- Line 384: author is encouraged to define the seasons and survey period more clearly throughout the manuscript
- The first paragraph of the discussion repeats the results. Can authors please re-phrase as an interpretation of results to tie into discussion points. Could try focusing on topic sentences and then backing up with a summary of result to back up point.
- Line 426: I wonder if other factors influence dolphin presence in an area in high tide, such as more water and therefore room to swim, rest, socialise (what was the water depth in these area), also there may be benefits acoustically during higher tide, less rapid attenuation of calls and therefore more successful fishing? Just thoughts – perhaps the author could explore some other potential hypothesis.
- Water depth would be a good variable to include in analysis
- Line 437: why are other species highly likely to occur in the summer period? Can you provide references?
- Author should identify the limitations with data and room for improvement i.e. bias with observers and effort and level of training.
