# Supplementary material for: Spatial and temporal variation in the occurrence of bottlenose dolphins in the Chesapeake Bay, USA, using citizen science sighting data
Source: PLoS One. 2021 May 18;16(5):e0251637. doi: 10.1371/journal.pone.0251637 (PMC8130941; doi:10.1371/journal.pone.0251637)
Supplement: Attachment — Submitted filename: ResponseToReviewers_2.docx [file pone.0251637.s004.docx]

PONE-D-20-27115R1

*Spatial and temporal variation in the occurrence of bottlenose dolphins in the Chesapeake Bay, USA, using citizen science data*

Ms. Lauren Kelly Rodriguez

**1) Thank you for stating in your methods that: "A C-POD (Chelonia Wildlife Acoustic Monitoring,** [**www.chelonia.co.uk**](http://www.chelonia.co.uk/)**) was deployed in the middle Bay at the mouth of the Potomac River (37.914 °N, 76.258 °W) from May through October during each study year (Fig 1a)."**

**To comply with PLOS ONE submissions requirements for field studies, please provide the following information in the Methods section of the manuscript and in the “Ethics Statement” field of the submission form (via “Edit Submission”):**

**a) Provide the name of the authority who issued the permission for each location (for example, the authority responsible for a national park or other protected area of land or sea, the relevant regulatory body concerned with protection of wildlife, etc.). If the study was carried out on private land, please confirm that the owner of the land gave permission to conduct the study on this site.**

The C-POD used in this study was not deployed in a park, sanctuary, within a navigational channel, or in any other protected area which would require regulatory approval. Additionally, the study relied solely on passive data collection and therefore does not require a permit under the U.S. Endangered Species Act, the Marine Mammal Protection Act, or any state's equivalent natural resource law.

**b) For any locations/activities for which specific permission was not required, please**

**- i. state clearly that no specific permissions were required for these locations/activities, and provide details on why this is the case**

**- ii. confirm that the field studies did not involve endangered or protected species**

This study involved the *passive* acoustic monitoring of the protected species, bottlenose dolphins and thus, no specific permissions were required.

**c) For vertebrate studies only, please provide the following additional information:**

**- i. Full details of collection and sampling methods, including method of sacrifice if applicable**

**- ii. State whether the vertebrate work was approved by an Institutional Animal Care and Use Committee (IACUC) or equivalent animal ethics committee. If no approval was obtained, please explain why it was not required.**

**- iii. State clearly whether all sampling procedures and/or experimental manipulations were reviewed or specifically approved as part of obtaining the field permit.**

While the goal of this study was to study vertebrate ecology, the data collection solely utilized a passive acoustic recorder. There was physical interaction with, collection of, or sacrifice of marine mammals or other vertebrates for this research. Therefore, no IACUC approval was required, nor were permits or authorizations under any natural resources law.

**2) We note that you have resubmitted new copies of Figs 1, 4, and 5, but you have not addressed the copyright concerns for these images. Please respond to our concerns, listed again below:**

Figures 1, 4, and 5 utilize the World Light Gray Reference basemap available with ArcGIS 10.6. This basemap was compiled by Esri using HERE data, Garmin basemap layers, OpenStreetMap data, GIS community data, and Esri basemap data. As indicated by the Esri Master License Agreement (<https://www.esri.com/content/dam/esrisites/en-us/media/legal/ma-translations/english.pdf>, <https://downloads2.esri.com/arcgisonline/docs/tou_summary.pdf>), this basemap is allowed to be printed in academic publications and does not need further permission from the copyright holder. We have included additional attribution to Esri and its data providers in the captions for Figs 1, 4, and 5.

**3) We note that your manuscript is not formatted using one of PLOS ONE’s accepted file types. Please reattach your manuscript as one of the following file types: .doc, .docx, .rtf, or .tex (accompanied by a .pdf).**

**If your submission was prepared in LaTex, please submit your manuscript file in PDF format and attach your .tex file as “other.”**

Our manuscript file has been reuploaded as a .docx file labeled “Manuscript.docx”.

**4) Please amend the title either on the online submission form or in your manuscript so that they are identical.**

We have ensured that each part of the online submission form as well as all uploaded copies of the manuscript share identical titles.
