# Supplementary material for: Spatial and temporal variation in the occurrence of bottlenose dolphins in the Chesapeake Bay, USA, using citizen science sighting data
Source: PLoS One. 2021 May 18;16(5):e0251637. doi: 10.1371/journal.pone.0251637 (PMC8130941; doi:10.1371/journal.pone.0251637)
Supplement: Attachment — Submitted filename: ResponseToReviewers_3.docx [file pone.0251637.s005.docx]

PONE-D-20-27115R1

*Spatial and temporal variation in the occurrence of bottlenose dolphins in the Chesapeake Bay, USA, using citizen science data*

Ms. Lauren Kelly Rodriguez

1. **Please review your reference list to ensure that it is complete and correct. If you have cited papers that have been retracted, please include the rationale for doing so in the manuscript text, or remove these references and replace them with relevant current references. Any changes to the reference list should be mentioned in the rebuttal letter that accompanies your revised manuscript. If you need to cite a retracted article, indicate the article’s retracted status in the References list and also include a citation and full reference for the retraction notice.**

The manuscript’s reference list was reviewed to ensure all appropriate citations were included. One reference (#23: R Core Team. R: A language and environment for statistical computing. R Foundation for Statistical Computing. 2017.) was added to the reference list in this version of the manuscript after being left out in prior versions.

*In-text line 195-198: To determine whether the acoustic detections and reported sightings showed similar trends in dolphin presence, a Spearman’s Rank Correlation test in R using the cor.test function was utilized [23].*

**Abstract:**

2. **Can the last sentence be broadened? Can the models can be used for predictive tool for species occurrence in important cetacean habitat, including Chesapeake Bay?**

The scope of this paper is to use our Generalized Additive Models as a predictive tool for this species’ (bottlenose dolphins, *Tursiops truncatus*) occurrence in the Chesapeake Bay. As it is the first paper of its kind in this urban region, it’s noteworthy information to share these models with management and other regulatory agencies in the area. However, the response of bottlenose dolphins to the environmental conditions of this Mid-Atlantic system may differ from the response of dolphins in other regions that have very different temperature and salinity regimes, such as that of the Gulf of Mexico or off California. Therefore, we only recommend using these models for predictive capabilities in the Chesapeake Bay, although similar statistical modeling approaches could be used in other locations.

**Methods/Results:**

3. **The bias and potential error in location data is explained in the response to reviewers, but it is not clear where it is described in the manuscript text. Please update the manuscript to include a description of the biases raised in the first review. Is there a protocol that requires the citizen scientists to report the sighting at the location of sighting? What measures are in place to prevent them from carrying on with their day and then reporting the sighting at a different time and location?**

The sighting location can be based on the device’s GPS location if reported at the time of the sighting or by selecting a point on the map if the user enters the sighting afterwards or they saw the dolphins from the shore. If the sighting location is plotted on land or if it does not match the description entered, the sighting was not confirmed and not included in our final database for the analyses. We have added this information on lines 89-98, 120-126, and 148-152.

*In-text lines 89-98: To account for variability in environmental conditions throughout the estuary, and because the potential location error in the sightings reported by our citizen scientists was unknown, we spatially divided the Chesapeake Bay in this study into three latitudinally-equal segments (Upper, Middle, Lower) with the Lower segment being the one closest to the Atlantic Ocean (Fig 1).*

*In-text lines 120-126: Each sighting report entered into Chesapeake DolphinWatch required the location of the sighting, which could be obtained directly from the user’s device or by the user selecting a point on a map. The time and date of the sighting were also recorded. A section for additional details on the sighting allowed users to include a description and/or pictures and videos of what they observed (Fig 2). Users could enter this information during the sighting or afterwards.*

*In-text lines 148-152: Users periodically enter sightings with insufficient information to be considered a true sighting and in these instances the data remained in the application but are deemed “unconfirmed”. Sighting locations that did not match the entered description or that occurred on land were not confirmed. Unconfirmed sightings were not included in this analysis.*

4. **More clarity should be provided in the manuscript on the acceptance criteria for ‘accepting’ sightings. I understand that supplementary material is provided, but I think the manuscript would benefit from a short description.**

We have included additional detail on the acceptance criteria that we used to confirm citizen science dolphin sightings in the manuscript from lines 142-152.

*In-text lines 142-152: For a reported sighting to be confirmed as a true sighting, it must meet the following requirements: 1) there is a photo or video of the encounter to accompany the sighting details provided and the location of the sighting is plotted in the water, or 2) there is a description of the sighting event. Some sightings from “trusted application users,” a small group of people who were in contact with our team and regularly submitted reliable sighting reports, were confirmed even if no description or photographic evidence was included. Users periodically enter sightings with insufficient information to be considered a true sighting, and in these instances the data remain in the application but are deemed “unconfirmed”. Sighting locations that did not match the entered description or that occurred on land were not confirmed. Unconfirmed sightings were not included in this analysis.*

5. **Table 1: total sightings reports for 2017-2019 add up to 2,907. This is inconsistent with the text that states that a total of 2,759 sightings were reported. Similarly Table 1 reports a total of 1,788 confirmed sightings and text states 1,870. Please cross check all values in text and figures throughout the manuscript.**

Thank you for catching this inconsistency. We have re-checked and updated all summary statistics throughout the manuscript.

**Discussion:**

6. **What are the current threats to dolphins in the Chesapeake Bay? As a suggested future study it would be important for conservation management to understand the spatial overlap between dolphin occurrence and human activities i.e. boating, fishing, water quality (is there contaminated water?), is habitat degradation a risk?**

The introduction of this manuscript (lines 35-41) describes some of the threats to dolphins in the Chesapeake Bay including those from 1) naval operations, 2) recreational boating, and 3) commercial fishing/shipping operations. We have added sentences on lines 384-385 and 478-481 in the discussion to emphasize the importance of these anthropogenic threats and habitat types in and around the Chesapeake Bay.

*In-text lines 35-41: The Chesapeake Bay is a large, highly urbanized estuary along the Mid-Atlantic coast of the USA. The Bay is characterized by tourism, military activities, and shipping; all of which can disturb marine species [3]. The Chesapeake Bay is home to two of the largest shipping ports in the USA (in Baltimore, Maryland and Hampton Roads, Virginia) as well as the largest naval base in the world (in Norfolk, Virginia). Dolphins that frequent this area are likely to encounter noise and physical disturbance from recreational and commercial shipping as well as from naval training exercises.*

*In-text lines 384-385: Between regional naval operations, urban coastal construction, and recreational activity, the Chesapeake Bay is a relatively loud and crowded marine environment.*

*In-text lines 478-481: Because our models allow predictions of dolphin occurrence based on environmental conditions, these abiotic parameters can be used to determine when and where dolphins will occur in any time period. Our data and models could be used to study the potential overlap between dolphin occurrence and anthropogenic activities, such as vessel traffic and fishing. They could also be studied in relation to different habitat types that have been degraded or restored, such oyster reefs and submerged aquatic vegetation (underwater grasses).*

7. **Is there any current management protection for the cetaceans in the Bay? Can you provide some recommendations to management?**

The current management protection for bottlenose dolphins along the Mid-Atlantic region of the United States is the Marine Mammal Protection Act, which is not specific to the Chesapeake Bay. Prior to the launch of the Chesapeake DolphinWatch platform and publication of this paper, natural resource managers were not aware of, and therefore did not consider, the frequent and regular occurrence of bottlenose dolphins in the Chesapeake Bay. The recommendation that we have within this manuscript is that bottlenose dolphin presence, as a protected species, be incorporated in future Environmental Impact Statements, which are required for any construction or activity that may cause a major disturbance. This description has been expanded in the discussion on lines 457-475.

*In-text lines 457-475: The U.S. Marine Mammal Protection Act requires activities that may adversely affect marine mammals to receive permits. It also requires that the U.S. National Marine Fisheries Service (NMFS) ensure that all anthropogenic activities authorized by the Service have the least practicable adverse impact to local impacted marine mammals [40]. Previously, NMFS has not considered bottlenose dolphins as regular inhabitants of the Chesapeake Bay or required their inclusion within Environmental Impact Statements related to proposed developments and activities within the Bay that could cause harm or disturbance to the dolphins. When data is scarce, NMFS is required to take a precautionary approach, assuming that marine mammals would be present. This precautionary approach may result in increased costs or restrictions to activities, which ultimately do little to nothing to protect the animals. Our data and models on the spatiotemporal distribution of bottlenose dolphins should greatly assist in identifying times and locations that will minimize the impact to bottlenose dolphins and when mitigation measures would be most beneficial. For example, mitigations imposed on construction projects in the Chesapeake Bay in the month of October may be costly with little benefit provided to the population as most animals will have left the Bay by then whereas avoiding scheduling any potentially harmful activities in the peak months of June to August could have the most benefit.*

8. **Line 414-422: please revise this paragraph. The explanation of the precautionary principle doesn’t make sense to me.**

This paragraph has been revised for clarity.

*In-text lines 456-475: This study offers insight into the seasonal presence of dolphins within the Chesapeake Bay, which is crucial to the ecological management of this region. The U.S. Marine Mammal Protection Act requires activities that may adversely affect marine mammals to receive permits. It also requires that the U.S. National Marine Fisheries Service (NMFS) ensure that all anthropogenic activities authorized by the Service have the least practicable adverse impact to local impacted marine mammals [40]. Previously, NMFS has not considered bottlenose dolphins as regular inhabitants of the Chesapeake Bay or required their inclusion within Environmental Impact Statements related to proposed developments and activities within the Bay that could cause harm or disturbance to the dolphins. When data is scarce, NMFS is required to take a precautionary approach, assuming that marine mammals would be present. This precautionary approach may result in increased costs or restrictions to activities, which ultimately do little to nothing to protect the animals. Our data and models on the spatiotemporal distribution of bottlenose dolphins should greatly assist in identifying times and locations that will minimize the impact to bottlenose dolphins and when mitigation measures would be most beneficial. For example, mitigations imposed on construction projects in the Chesapeake Bay in the month of October may be costly with little benefit provided to the population as most animals will have left the Bay by then whereas avoiding scheduling any potentially harmful activities in the peak months of June to August could have the most benefit.*

9. **Line 416: should this be prohibited, not ‘permitted’? Please revise sentence.**

We have changed the wording of this sentence.

*In-text lines 457-459: The U.S. Marine Mammal Protection Act requires activities that may adversely affect marine mammals to receive permits.*

10. **Line 417: is practical effect the correct terminology here? I would think that ‘potential effect’ is more suitable.**

The correct terminology in this case is "least practicable adverse impact". We have changed the wording of this sentence to make it clearer.

*In-text lines 459-461:. It also requires that the U.S. National Marine Fisheries Service (NMFS) ensure that all anthropogenic activities authorized by the Service have the least practicable adverse impact to local impacted marine mammals [40].*

11. **Line 427: I think ‘determine’ should be replaced with ‘infer’ or ‘indicate’ when are where dolphins ‘may occur seasonally’.**

We agree that “infer” is a clearer describer than “determine”. We have made this change in the manuscript.

*In-text lines 479-481: Because our models allow predictions of dolphin occurrence based on environmental conditions, these abiotic parameters can be used to infer when and where dolphins will occur in any time period.*

12. **Last sentence: I think the last sentence could be more punchy.**

Thank you for this suggestion. We have added a sentence to emphasize the impacts of our research.

*In-text lines 485-494: This study, the first description of the spatiotemporal distribution of bottlenose dolphins within the Chesapeake Bay, provides a baseline from which future patterns of occurrence can be compared. Additional collection of acoustic and environmental data would provide context to the sightings made by citizen scientists and aid in determining the behaviors, including foraging, of dolphins in the Bay. This behavioral context would improve both the ecological understanding and management of bottlenose dolphins within the Chesapeake Bay. This study is the first description and model of the spatiotemporal distribution of bottlenose dolphins within the highly urbanized Chesapeake Bay. These findings can be used by resource managers to minimize the impacts of the many current and proposed anthropogenic activities in this region.*
